# Skeletal muscle in health and disease

**DOI:** 10.1242/dmm.042192

**Published:** 2020-02-06

**Authors:** Jennifer Morgan, Terence Partridge

**Affiliations:** 1 Dubowitz Neuromuscular Centre, UCL Great Ormond Street Institute of Child Health, 30 Guilford Street, London WC1N 1EH, UK; 2 National Institute for Health Research, Great Ormond Street Institute of Child Health Biomedical Research Centre, University College London, London WC1N 1EH, UK; 3 Center for Genetic Medicine Research, Children's National Medical Center, 111 Michigan Ave NW, Washington, DC 20010, USA

**Keywords:** Muscular dystrophy, Satellite cell, Skeletal muscle regeneration

## Abstract

Skeletal muscle fibres are multinucleated cells that contain postmitotic nuclei (i.e. they are no longer able to divide) and perform muscle contraction. They are formed by fusion of muscle precursor cells, and grow into elongating myofibres by the addition of further precursor cells, called satellite cells, which are also responsible for regeneration following injury. Skeletal muscle regeneration occurs in most muscular dystrophies in response to necrosis of muscle fibres. However, the complex environment within dystrophic skeletal muscle, which includes inflammatory cells, fibroblasts and fibro-adipogenic cells, together with the genetic background of the *in vivo* model and the muscle being studied, complicates the interpretation of laboratory studies on muscular dystrophies. Many genes are expressed in satellite cells and in other tissues, which makes it difficult to determine the molecular cause of various types of muscular dystrophies. Here, and in the accompanying poster, we discuss our current knowledge of the cellular mechanisms that govern the growth and regeneration of skeletal muscle, and highlight the defects in satellite cell function that give rise to muscular dystrophies.

## Abstract (precis)

**Summary:** The mechanisms of skeletal muscle development, growth and regeneration are described. We discuss whether these processes are dysregulated in inherited muscle diseases and identify pathways that may represent therapeutic targets.

## Introduction

Skeletal muscle is composed of linear arrays of multinucleated muscle fibres, each with a complex internal structure dedicated to the conversion of chemical to physical energy. These fibres are ‘end cells’, meaning that they cannot proliferate to expand or restore the population after damage. Instead, they are formed or repaired by fusion of a proliferation-capable population of precursor cells called myoblasts (see Glossary, Box 1). The sequence of transcription factor expression leading to differentiation in the precursor cell population of the main mammalian body musculature is a close reflection of that observed during initial muscle formation in the embryo and the enlargement of muscle fibres in the postnatal and juvenile stages of muscle growth, as well as that observed in muscle repair. However, the behaviour of the myogenic (Box 1) cells differs radically between these situations.

**Figure:**
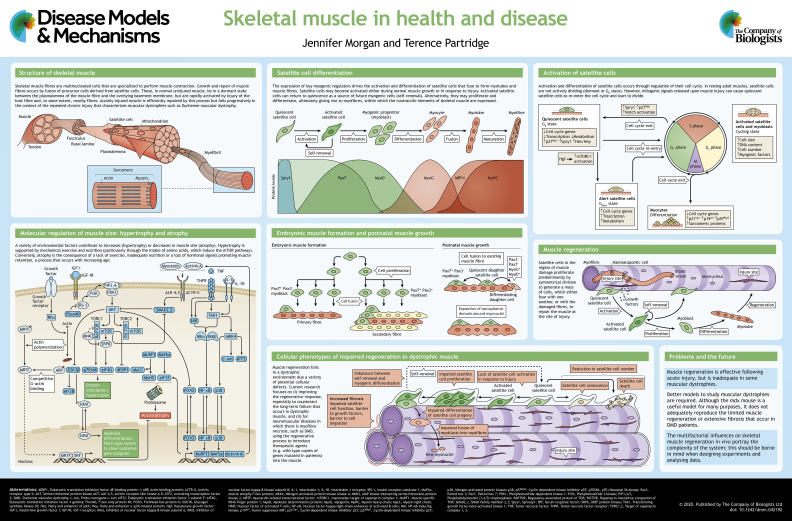

**Box 1. Glossary**

**Asymmetric division:** a cell division that produces daughter cells that have different fates (e.g. one stem cell and one differentiated cell).

**CTG expansion:** a mutation in which repeats of three nucleotides (trinucleotide repeats) increase in copy number until they cross a threshold above which they become unstable.

**DBA2/J, C57Bl/10 and 129/SVemst/J:** inbred mouse strains that differ in their genetic backgrounds.

***Dy/dy* mouse:** a model of laminin alpha-2 deficiency (MDC1A) that has a mutation in the *LAMA2* gene. This mouse has a moderate fibrotic and dystrophic phenotype (reviewed in Ng et al., 2012).

**Dysferlin:** a protein that is highly expressed at the sarcolemma of muscle fibres and is involved in repair of the sarcolemma.

**Dystroglycanopathy:** a muscular dystrophy caused by aberrant glycosylation of dystroglycan.

**Gamma-sarcoglycan (*Sgcg*)-null mouse model:** a model of Limb-girdle muscular dystrophy type 2C (LGMD2C).

**Genetic modifier:** a gene that affects the phenotypic and/or molecular expression of other genes.

***Large^myd^* mouse:** a model of congenital muscular dystrophy type 1D (MDC1D). Dystroglycan glycosylation is defective in these mice as a result of a mutation in like-acetylglucosaminyltransferase (LARGE), a glycosyltransferase.

**Mdx mouse:** X-linked muscular dystrophy mouse model of DMD. Has a mutation in exon 23 of the *Dmd* gene.

**Muscle precursor cell:** any cell that is predetermined to differentiate into skeletal muscle.

**Myoblast:** the progeny of satellite cells.

**MyoD:** myoblast determination protein 1, a myogenic regulatory factor.

**Myogenic:** originating in, or produced by, muscle cells.

**Niche:** a stem cell niche is an interactive structural unit, organized to facilitate cell-fate decisions in a proper spatiotemporal manner (Moore and Lemischka, 2006).

**Satellite cell:** skeletal muscle stem cell, located between the basal lamina (the internal layer of the basement membrane) and sarcolemma (cell membrane) of a muscle fibre. A satellite cell expresses PAX7 and is quiescent in normal adult muscle.

**Symmetric cell division:** a cell division that produces daughter cells that have the same fate (e.g. two stem cells, or two differentiated cells).

**Utrophin:** a cytoskeletal protein that has some structural and functional similarities to dystrophin.

Here, we briefly discuss skeletal muscle formation, growth and repair, with particular reference to muscular dystrophies. Most of the data behind these descriptions are derived from studies in animal models, mainly rodents, or from *in vitro* models of myogenesis. The relationship between the human condition of interest and the animal models requires careful consideration (Partridge, 2013). Likewise, while *in vitro* or *ex vivo* models of myogenesis are the source of much of the molecular biological data on myogenesis, they do not reproduce the interactions with the cellular, matrix and systemic features of the *in vivo* environment that tune the process of myogenesis to the physiological needs of the animal as a whole. Thus, the applicability of knowledge for disease treatment gained from the above models should be treated with reserve.

## Initial muscle fibre formation

Initial muscle fibre formation has predominantly been studied in the limb. During initial myogenesis in the embryonic muscle anlagen, precursor cells proliferate to form compact groups, within which individual cells fuse together in longitudinal arrays to form multinucleated fibres (see poster). This occurs in phases, beginning with a synchronous fusion of cells expressing the paired box transcription factors Pax3 and Pax7 across the whole length of the newly emerging muscle anlagen to form primary muscle fibres (Lee et al., 2013), which act as a scaffolding for subsequent rounds of fibre formation. In mice, a second subset of Pax3^+^, Pax7^−^ myogenic cells associate and align with the primary fibres. They fuse sequentially with one another, beginning in the middle of the fibre and progressing towards the two ends, to form secondary fibres (Lee et al., 2013) (see poster). In large mammals, a tertiary and even a quaternary phase of myogenesis may occur, although the evidence is uncertain (Edom-Vovard et al., 1999; Bröhl et al., 2012).

## Growth of muscle fibres

In mice, neoformation of muscle fibres ceases by birth. Muscle growth occurs by a combination of the progressive addition of myonuclei to each fibre and the expansion of the sarcoplasmic domain around each myonucleus (see poster). In mice, the addition of new myonuclei is largely accomplished by 3-4 weeks of age, and entails both the proliferation and fusion of satellite cells (Box 1). Between 2-3 weeks of age, each mouse extensor digitorum longus (EDL) myofibre increases in myonuclear number from ∼100 to ∼200 myonuclei (Duddy et al., 2015; White et al., 2010). This corresponds to one satellite cell fusion every 2 h and is accomplished by around 5-10 satellite cells per fibre (Duddy et al., 2015; White et al., 2010). Muscle growth beyond 4 weeks of age continues predominantly by an increase in the sarcoplasmic territory around each myonucleus but also involves the addition of myonuclei at about one tenth of the pre-3-week rate, again brought about by the action of a small number of satellite cells per fibre (Duddy et al., 2015).

## Models of muscular dystrophies

Skeletal muscle development, muscular dystrophies and muscle regeneration have been studied in different *in vivo* models (Table 1). Of these, the mdx mouse (Box 1) has been the most used, and is thus the source of the most comprehensive set of detailed pathological data; however, its mild clinical course (Bulfield et al., 1984) has raised concerns about its use as a model of Duchenne muscular dystrophy (DMD) in humans. There is a marked difference between the pathology of dystrophin-deficient mice and humans, possibly due to the far greater growth span, larger size and greater loading of muscles in humans than in mice (Grounds and Shavlakadze, 2011). The primary pathology is severe in mdx mice, but is counteracted by robust skeletal muscle regeneration. However, severity is increased in the context of mutations in other genes that affect myoblast proliferation or myofibre stability, such as those that affect telomere length (Sacco et al., 2010) (Yucel et al., 2018), cause a lack of utrophin (Box 1) (Deconinck et al., 1997) or myoblast determination protein 1 (MyoD; also known as Myod1; Box 1) (Megeney et al., 1996), or lead to the deletion of the mouse cytidine monophosphate-N-acetylneuraminic acid hydroxylase (*Cmah*) gene (Chandrasekharan et al., 2010) (reviewed in Rodrigues et al., 2016).

**Table 1.**
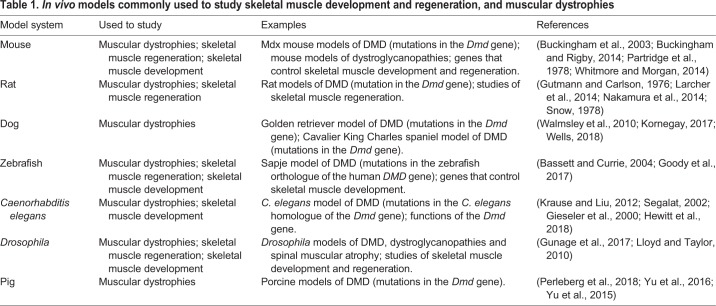
***In vivo* models commonly used to study skeletal muscle development and regeneration, and muscular dystrophies**

The more severe disease seen in golden retrievers with muscular dystrophy is widely regarded as a closer model of DMD pathology than the mdx mouse and an important intermediate for preclinical research (Kornegay, 2017), but the cost of experiments involving dogs limits their value for fundamental research. Recently developed rat models (Larcher et al., 2014; Nakamura et al., 2014) have yet to be sufficiently described to be fully assessable as experimental models. Fish and invertebrate models offer good access to tools that facilitate the investigation of the genetic and molecular biological aspects of the disease process (Table 1), but the inflammatory responses to damage differ substantially from those in mammals, meaning that these models are of limited value to study the inflammatory aspects of human disease.

## Skeletal muscle regeneration

### Cellular mechanisms of muscle regeneration

Muscle fibres respond to minor damage with limited immediate repair mechanisms that reseal the muscle fibre's surface membrane (Barthélémy et al., 2018; Horn et al., 2017). Conversely, repair and replacement of irreversibly damaged fibres is achieved by activation of muscle precursor cells (Box 1), which proliferate, move to the area of damage, and fuse with one another and with the surviving segments of damaged muscle fibres (see poster). This latter process does not always perfectly align the surviving fibre stump with the newly forming repair segment, with the result that many fibres become branched after regeneration (Blaveri et al., 1999) (Partridge and Morgan, 2014) and progressively so in the context of a chronic myopathy such as muscular dystrophy (Duddy et al., 2015).

Skeletal muscle regeneration is mediated largely, if not exclusively, by satellite cells (Lepper et al., 2011; Sambasivan et al., 2011; Yamamoto et al., 2018). These cells are normally quiescent in undamaged adult muscle, but become activated in response to injury. They express receptors for growth factors [e.g. fibroblast growth factor receptor 2 (Kästner et al., 2000)], which drive their proliferation upon release of growth factors from damaged muscles or inflammatory cells. This very rapid activation is attributed to the release of RNA coding for the myogenic transcription factor Myf5 from cytoplasmic granules (Crist et al., 2012) and involves a rapid onset of expression of MyoD, with satellite cell proliferation beginning 24-36 h later (Cornelison and Wold, 1997; Zammit et al., 2004). It is hypothesized that some cells contribute to regeneration by fusing with one another and with damaged muscle fibres, while others, distinguished by cessation of MyoD expression, become quiescent and re-enter the satellite cell pool (Zammit et al., 2004). Most of these ideas are based on the study of satellite cells adherent to myofibres, which can be isolated from muscle and subsequently used as an *in vitro* model of regeneration. But it is becoming increasingly clear that other features of the injury environment play important roles in modulating the sequence of regeneration events. Investigation of the fate of satellite cells (Cousins et al., 2004; Robertson et al., 1990; Tierney et al., 2018a; Webster et al., 2016), myofibre necrosis (Chrzanowski et al., 2017; Filareto et al., 2018) and myofibre regeneration (Baudy et al., 2011) in the context of inflammation (Martinez et al., 2015) in *in vivo* models are more informative as to the extent of participation of other cells and of the intercellular environment. Recently available markers have shown a complex diversity of myogenetic clones that remain stable during growth and ageing of normal muscle (Tierney et al., 2018b), implying that asymmetric division (Box 1) of a stem cell compartment is a major component of muscle formation and maintenance. During regeneration, however, myogenic clones increase in size and diminish in their complexity, suggesting that, in this process, muscle cells expand predominantly by symmetric cell division (Box 1) of committed cells.

It is increasingly apparent that repair of muscle is a complex collaborative activity, involving several different cell types in addition to satellite cells (Wosczyna and Rando, 2018). Of these, the macrophage (Chazaud et al., 2003; Tidball, 2017) has become prominent, and its effects on the overall repair process are phased. The initial ‘pro-inflammatory’ macrophage population, which is envisaged to act predominantly in the resorption of damaged tissue, is subsequently transformed into, or succeeded by, a more ‘pro-regenerative’ type of macrophage, which secretes cytokines that facilitate the myogenic functions of satellite cells (Kharraz et al., 2013; Saclier et al., 2013b; Tidball et al., 2014; Tidball and Villalta, 2010). The activities of resident fibro-adipogenic cells also influence the balance between fibrosis and myogenesis during the repair process in damaged muscle (Joe et al., 2010; Uezumi et al., 2011) and strongly affect the degenerative mechanisms in dysferlin (Box 1)-deficient muscle (Hogarth et al., 2019). Such discoveries have also revived interest in the changes in structural components of muscle associated with chronic inflammation, which are likely to impact cell mobility and the distribution of cytokines. Fibrosis is an issue in muscular dystrophies, posing a physical barrier to cells and altering muscle stiffness, which can affect satellite cell function (reviewed in Smith and Barton, 2018) (see poster). Furthermore, non-muscle cells, such as fibroblasts (Murphy et al., 2011; Fry et al., 2017; Mackey et al., 2017), and the interactions between satellite cells and cells of the microvasculature (Abou-Khalil et al., 2010; Mounier et al., 2011; Saclier et al., 2013a; Verma et al., 2018), are also involved in mediating the inflammatory response and in promoting satellite cell proliferation and differentiation. The complexity of the cellular interactions in skeletal muscle is too extensive and intricate to be effectively modelled in terms of individual cellular processes. Thus, muscle is perhaps better regarded as an ecosystem within which each of the component parts contributes to a homeostasis that may be disturbed by extreme pathological processes or genetic defects.

There is evidence that cells other than satellite cells contribute to skeletal muscle regeneration, e.g. Twist2-dependent progenitors (Liu et al., 2017), pericytes (Dellavalle et al., 2011; Dellavalle et al., 2007; Meng et al., 2011; Meng et al., 2015; Meng et al., 2016) and CD133^+^ (also known as PROM1^+^) cells (Negroni et al., 2009; Torrente et al., 2004; Meng et al., 2014; Meng et al., 2015), but failure of regeneration in the absence of satellite cells questions practical role of these additional cells in this process (Lepper et al., 2011; Sambasivan et al., 2011; Yamamoto et al., 2018).

### Effect of gene mutations on satellite cell function

Skeletal muscles regenerate to different extents in different mouse dystrophy models. For example, *Large^myd^* (also known as *Large1^myd^*) mice (Box 1) show little regeneration (Bröhl et al., 2012; Almeida et al., 2016) in contrast to the extensive regeneration in the muscles of mdx mice (Almeida et al., 2016). In the context of different genetic defects, it is difficult to determine the extent to which differences in regenerative outcome are influenced by variation in the pattern and extent of fibre degeneration. Interpretation is further complicated by the fact that the perturbation or loss of any of the various genes involved in muscle regeneration may have pleiotropic effects across a number of tissues.

If a defective gene is normally expressed in satellite cells or their progeny, then these cells can be directly affected by the genetic defect. For example, although dystrophin is an important structural protein within skeletal and cardiac muscle and brain, it is also expressed in newly activated satellite cells (Zhang and McLennan, 1994), and its lack in the mdx mouse has been demonstrated to disturb asymmetric division in mdx satellite cells *ex vivo* (Dumont et al., 2015), with the conjecture that this would deplete the numbers of fusion-competent satellite cells (Dumont et al., 2015) and impair muscle regeneration. However, this prediction conflicts with the fact that dystrophic muscles appear to form normally in all mammalian models of DMD and that mdx limb muscles regenerate very well in response to intrinsic myofibre necrosis [doubling their myonuclear content over the first 3 months of the disease (Duddy et al., 2015)] and in response to experimental injury, even in very old muscles (Boldrin et al., 2015). Such *in vivo* observations argue against any intrinsic problem with myogenesis of dystrophic myoblasts and satellite cells.

Defects in genes that are normally expressed only in the muscle fibre or connective tissue may have indirect effects on satellite cell function. For example, there is no innate defect in the proliferative ability of satellite cells in the *dy/dy* mouse (Box 1) model of laminin alpha-2 chain/merosin-deficient congenital muscular dystrophy (in which skeletal muscles rapidly degenerate, but regenerate poorly) when they are removed from their niche (Box 1) (Ontell et al., 1992). However, in mouse models of dystroglycanopathy (Box 1) (Ross et al., 2012) and collagen VI deficiency (Urciuolo et al., 2013), the effects of niche defects on satellite cell dysfunction have yet to be fully delineated. Collagen VI deficiency directly affects basement membrane structure, but may also indirectly effect satellite cell behaviour as a consequence of a series of degeneration/regeneration events: in each event, a myofibre undergoes necrosis and it is either repaired by satellite cells, or is replaced by a newly regenerated myofibre. A myofibre may undergo more than one of these degeneration/regeneration events. When each occurs, either a new basal lamina forms within the old one, or the old basal lamina may be removed and replaced by a newly formed basal lamina (Gulati et al., 1983; Vracko and Benditt, 1972). Furthermore, mutations in different components of the dystrophin-associated protein complex (DAPC), which spans the myofibre plasma membrane (reviewed in Gao and McNally, 2015), cause different muscular dystrophies (reviewed in Whitmore and Morgan, 2014) (Table 1). The extent to which members of the DAPC are expressed in satellite cells (Cohn et al., 2002), and whether their expression within satellite cells is an important component of satellite cell function (Dumont et al., 2015), remains unresolved.

### Satellite cell defects in muscular dystrophies

Different muscular dystrophies give rise to a variety of defects within satellite cells (Table 2; see poster) (Bigot et al., 2009; Thornell et al., 2009; Beffy et al., 2010; Castets et al., 2011; Logan et al., 2011; Boyden et al., 2012; Di Gioia et al., 2017; Feichtinger et al., 2019). Skeletal muscle in different parts of the body is differentially affected by muscular dystrophies (reviewed in Randolph and Pavlath, 2015), which has been suggested to result from differences in the type and/or the function of satellite cells within different muscles. For example, extraocular muscles are spared in several muscular dystrophies, possibly due to intrinsic developmental differences between extraocular and other muscles (reviewed in McDonald et al., 2015). Furthermore, extraocular muscles contain more satellite cells than hindlimb muscles (Kallestad et al., 2011), and satellite cells from extraocular muscles have a greater proliferative and regenerative capacity than those from limb and diaphragm muscles (Stuelsatz et al., 2015). In contrast, satellite cells of limb and masseter muscle origin contribute similarly to muscle regeneration on transplant into a permissive muscle environment, despite the fact that satellite cells from EDL and masseter muscles have different gene expression profiles, and masseter satellite cells usually proliferate more and differentiate later than those from EDL (Ono et al., 2010).

**Table 2.**
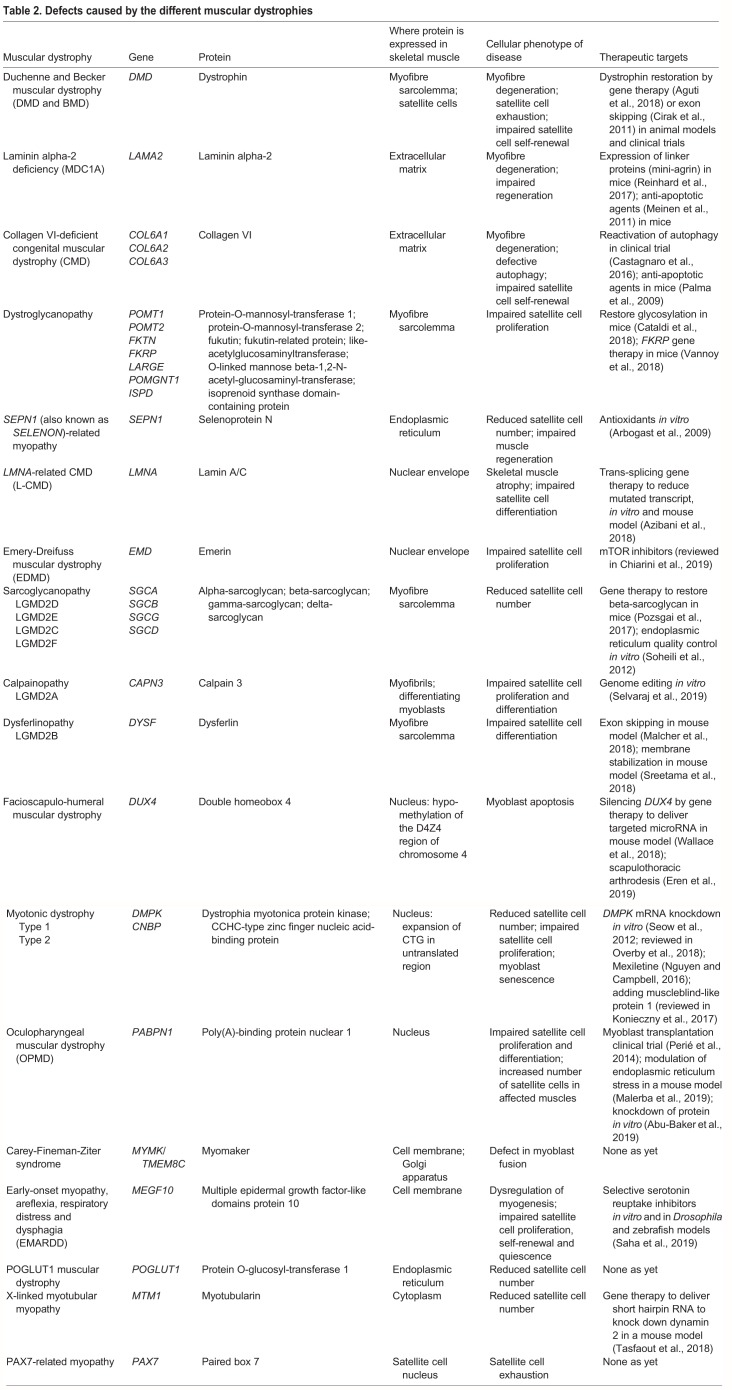
**Defects caused by the different muscular dystrophies**

Satellite cell number is also affected in muscular dystrophies, although there is contradictory evidence for their loss in DMD or mdx mouse muscle (Bankolé et al., 2013; Boldrin et al., 2015; Jiang et al., 2014; Kottlors and Kirschner, 2010). The denominator used for their quantification, i.e. loss of satellite cells per myofibre, per myonucleus, per total area or per muscle fibre area, may play some part in this. Satellite cell number is higher in pharyngeal muscles than in limb muscles of oculopharyngeal muscular dystrophy patients (Gidaro et al., 2013), and some of these PAX7^+^ cells lie outside the satellite cell niche, suggesting a problem with the niche itself, but the implications of these higher satellite cell numbers are unclear. Recessive mutations in the protein *O*-glycosyltransferase 1 (*POGLUT1*) gene are associated with decreased Notch signalling and patients have fewer quiescent satellite cells (Servián-Morilla et al., 2016), suggesting a critical role of POGLUT1 in the maintenance of the satellite cell pool. In normal human muscle, satellite cell numbers diminish with age (Sajko et al., 2004). In muscular dystrophies, age-related decreases in satellite cell number can be compounded by the chronic pathology and may occur by different mechanisms (e.g. by proliferative exhaustion). There is a reduction in satellite cell number in a mouse model of recessive selenoprotein 1-related myopathy (Castets et al., 2011), and their numbers are also attenuated in X-linked myotubular myopathy by a combination of apoptosis and reduced proliferation (Lawlor et al., 2012).

Lastly, genetic background also has a profound effect on muscle regeneration. Skeletal muscle pathology of the gamma-sarcoglycan (*Sgcg*)-null mouse model (Box 1) of limb-girdle muscular dystrophy (LGMD) is worse in a DBA2/J than a 129/SVemst/J genetic background (Fukada et al., 2010; Heydemann et al., 2009). Likewise, the DBA2/J background is associated with a worse mdx pathology than the C57Bl/10 background (Fukada et al., 2010; Coley et al., 2016; van Putten et al., 2019) (Box 1). Furthermore, genetic modifiers (Box 1) in DMD and facioscapulohumeral muscular dystrophy affect membrane-associated proteins that may preserve muscle fibres against degeneration (reviewed in Hightower and Alexander, 2018).

The quality of regeneration of dystrophic skeletal muscle is subject to ongoing discussion. Regenerated muscle fibres in normal, injured and mdx mouse muscles are invariably branched (Bourke and Ontell, 1984; Ontell, 1986; Pichavant and Pavlath, 2014). There is an association between the extent of branching and the vulnerability to contraction-induced injury in older fast-twitch muscles in mdx mice (Chan et al., 2007). Myofibre branching is a major factor in the hypertrophy of mdx muscle (Faber et al., 2014), but its direct effect on muscle strength is difficult to determine.

The main messages from this research are that satellite cells are dysfunctional in many chronic muscle diseases, and that this is compounded by increasing age. There is some debate as to whether myogenic deficit in any particular case is intrinsic to the satellite cells themselves or to the influence of the environment. The concept of epigenetic switching of cell function, by signalling from extracellular sources, now blurs this distinction. Whether ageing is intrinsic to the cells or is a reflection of the cellular response to the ageing environment is under debate.

## Hypertrophy of skeletal muscle

Muscle size is greatly influenced by the functional demands made on it, with atrophy being associated with disuse or underuse, while heavy use, particularly under high loads, promotes hypertrophy (Murach et al., 2017). Interestingly, although the molecular mechanisms and pathways associated with atrophy and hypertrophy are well described (reviewed in Egerman and Glass, 2014; Schiaffino et al., 2013) (see poster), the cellular mechanisms involved remain to be fully ascertained.

Conditional Cre ablation of satellite cells has led to mixed results and views on the role of satellite cell participation in the muscle growth response to overload that results from ablation of synergistic muscles (Egner et al., 2016; Lee et al., 2012; Murach et al., 2017). Among the best-described molecular mechanisms behind the control of muscle size are the insulin-like growth factor 1, transforming growth factor beta and myostatin signalling pathways (reviewed in Lee, 2004; Chen et al., 2016; Timmer et al., 2018) (see poster). Inhibition of myostatin has a dramatic effect on muscle size (Lee and McPherron, 2001), although the cellular mechanisms involved are uncertain. A number of investigations have implicated suppression of satellite cell function as a causative mechanism of myostatin action (e.g. McCroskery et al., 2003); however, studies on the myostatin-null mouse have demonstrated no evidence of this (Amthor et al., 2009; Wang and McPherron, 2012).

## Potential therapies for muscular dystrophies

Because defects in regeneration play a key role in muscular dystrophies, they have widely been considered as a potential target for improvement of therapy in DMD, either by preventing or reducing myofibre necrosis (Morgan et al., 2018) and/or increasing muscle regeneration (Table 1). This line of thought has been pursued from two different angles. First, it has been proposed to use myoblast fusion during the repair of damaged muscle fibre as vectors for introducing therapeutic genes, e.g. normal copies of the mutant gene, into the repaired muscle fibres (Partridge et al., 1989). However, this approach would only be suitable for a neuromuscular disease in which there is myofibre necrosis (e.g. in DMD) and requires a high success rate of myoblast transplantation, which has not been achieved in any preclinical research projects or in the clinical trials conducted to date (reviewed in Skuk and Tremblay, 2015). The main reason for this poor grafting efficiency is the massive loss of cells within hours of their intramuscular transplantation (Beauchamp et al., 1999; Fan et al., 1996; Guerette et al., 1997; Skuk and Tremblay, 2017), the cause of which remains unexplained and is a clear target for further research. Second, ineffective muscle repair, which is a feature of DMD, at least in the later stages of the disease, is an obvious target for improvement but the causal bases of this phenomenon remain poorly understood. Because the standard mdx mouse does not reproduce this poor regenerative response, a better model of this aspect of the disease – perhaps the DBA/2J mdx mouse (van Putten et al., 2019) – is required.

## Conclusions

Our knowledge of the molecular mechanisms behind muscle growth and repair has flourished in recent years but our insight into the cellular mechanisms involved in myogenic processes lags greatly behind. The fundamental model of the molecular control of myogenesis in vertebrate limb muscles via a cascade of transcription factors has largely been derived from developmental myogenesis and tissue culture models of adult myogenesis. However, the role of these proteins in the radically different *in vivo* conditions of neomyogenesis in the embryo and muscle fibre growth, hypertrophy and muscle regeneration in adult muscle has yet to be satisfactorily reconciled. It is possible, for instance, to make a major distinction between those occasions on which the process of satellite cell proliferation is followed by mass cell fusion, e.g. during embryonic neomyogenesis and regeneration of adult muscle, in contrast to the slower continuous proliferation of satellite cells and immediate fusion of committed daughter cells during the growth of fibres in postnatal muscle.

Satellite cells are dysfunctional in many muscular dystrophies. Defective satellite function will have to be addressed, possibly in combination with strategies targeting muscle fibres (e.g. gene therapy or exon skipping to restore dystrophin), to more fully improve muscle performance in patients.
